# Sociophonetic Variation in Vowel Categorization of Australian English

**DOI:** 10.1177/00238309231198520

**Published:** 2023-10-13

**Authors:** Debbie Loakes, Josh Clothier, John Hajek, Janet Fletcher

**Affiliations:** School of Languages and Linguistics, The University of Melbourne, Australia

**Keywords:** perception, sociophonetics, Australian English, regional variation

## Abstract

This study involves a perceptual categorization task for Australian English, designed to investigate regional and social variation in category boundaries between close-front vowel contrasts. Data are from four locations in southeast Australia. A total of 81 listeners from two listener groups took part: (a) so-called mainstream Australian English listeners from all four locations, and (b) L1 Aboriginal English listeners from one of the locations. Listeners heard front vowels /ɪ e æ/ arranged in 7-step continua presented at random. Varied phonetic contexts were analyzed, with a focus on coda /l/ because of a well-known prelateral merger of /e æ/ through mid-vowel lowering (e.g., *celery-salary*) reported to occur in some communities in this part of Australia. The results indicate that regional variation in Australian English is evident in perception. In particular, merging of /el/-/æl/ is shown to occur in the southernmost regions analyzed, but rarely in the northern regions of the geographical area under investigation. Aside from regional variation observed, age was also a factor in how participants responded to the task: older speakers had more merger than younger speakers in many locations, which is a new finding—previously, the merger was thought to be increasing in frequency over time, yet here we see this in only one location. Aboriginal English listeners also responded differently when compared with mainstream Australian English listeners. By analyzing the perception results across a variety of regional locations, with data from two different Australian social groups in the same location, this study adds a new dimension to our understanding of regional and social variations in Australian English.

## 1 Introduction

Analysis of sociophonetic variation in perception has been emerging as an area of study that gives linguists new and complex insights into the ways in which language users differ from one another in their understanding and processing of the patterning of speech variants (e.g., discussions in Campbell-Kibler, 2010; Drager, 2010; Foulkes, 2010; Hay & Drager, 2007; Jannedy & Weirich, 2014). Perception may be defined in various ways in different academic fields and subfields, and the way we use the term *perception* here is “the processes engaged when people are exposed to external stimuli, in this case linguistic material, and extract information from it” (Campbell-Kibler, 2010, p. 378). In her book *Native Listening*, Cutler (2012) draws on a rich history of studies in this area, describing the view that speech perception is based on the decoding of input, which is essentially a complex and ultimately probabilistic, experience-based, endeavor. Cutler (2012, p. 1) succinctly explains that “. . .listening to speech is so easy (when it is easy), or so hard (when it is hard), because it depends so much on our previous experience of listening to speech.”

In this study, both regional and social variations in perception are analyzed through the results of a phonetic categorization experiment carried out in one of the largest cities in Australia (Melbourne [ML]) and in three regional towns (Warrnambool [WN], Mildura [MI], and Albury-Wodonga [AW])—all located in and around the state of Victoria, which is itself in southeastern Australia. The reason we have chosen specifically these four sites is to understand better the geographic limits of the /el/->/æl/ merger, which is known to be characteristic of at least some parts of Victoria (see especially Bradley, 2004; Cox & Palethorpe, 2004; Loakes et al., 2017; Schmidt et al., 2021). An example of how this merger surfaces can be seen is in the imperfect rhyme (which is a perfect rhyme if the merger is used) “The Prime Minister will *tell youse*, about your Australian *values*” penned by Melbourne born and based cartoonist, Michael Leunig, which appeared in the Melbourne-based newspaper *The Age* (Leunig, 2017, italics ours).^1^

In Warrnambool, the study goes deeper into the question of how a person’s social group influences their categorization of vowel stimuli by analyzing responses from Aboriginal and non-Aboriginal (“mainstream”) Australian English (AusE) listeners. This part of the experiment will allow us to determine whether listeners who are geographically aligned behave similarly in vowel categorization tasks, and/or whether groups who are socially different also respond differently, despite living in the same area.

Phonemic mergers are said to be one of the most prominent ways for language variation and change in processing to be observed (e.g., Campbell-Kibler, 2010). This is because people can behave differently from one another in perception before changes in production emerge (e.g., Kleber et al., 2011), and because of the consequent misalignment in production and perception which can emerge in speaker–listeners.

The merger of /el/-/æl/ also occurs in other Englishes around the world. For example, in New Zealand English, it appears to be complete, and is relatively well-studied (see, for example, Hay et al., 2013; Thomas & Hay, 2005). We have previously analyzed this particular merger as occurring due to hypo-correction for some listener groups in Australia (e.g., Loakes, Clothier, et al., 2014; Loakes et al., 2017; Loakes, Hajek, et al., 2014). Essentially, it appears that the lowering and retraction of the /e/ in the prelateral environment causes some ambiguity with vowel height, triggering some listeners to reinterpret /el/ as /æl/ (Loakes et al., 2017); as mentioned earlier the complexities of this phenomenon are still not well understood, however, and we aim to explore this further in the current study.

AusE is reported to have three main dialect types: Mainstream Australian English (MAE; which is also known as “Standard” AusE, and encompasses urban and rural speech), Aboriginal English (AbE; defined in more detail below), and Ethnocultural AusE (Cox & Fletcher, 2017, pp. 12–13), which encompasses the Englishes used by speakers with different non-Anglo-Celtic cultural heritage backgrounds. As explained by Cox and Fletcher (2017, p. 13) the term “Australian English” is used as a superordinate label for all of these varieties, and in line with these definitions, we use the terms “Mainstream Australian English” and “Aboriginal English” to talk about the two main varieties spoken by our participants. In MAE, there may also be significant variation that is often reported to occur along a cline from broad (more marked and localized) to cultivated (more educated, less locally marked) (Cox & Fletcher, 2017). In AbE, variation can also be extreme (e.g., Loakes et al., 2022; Mailhammer et al., 2020) ranging from acrolectal (more like MAE) to basilectal (more like a creole) (Butcher, 2008).

### 1.1 AbE in Australia

Due to social and political factors causing a rapid decline in the number of traditional Indigenous languages spoken in Australia (e.g., Malcolm, 2018), many people in Australia who identify as Aboriginal, speak AbE.^2^ This variety is structurally different from MAE used as the dominant variety by (typically) Anglo Australians in daily and institutional life (government, schools, media, etc.). It is also relatively poorly understood in mainstream culture, largely unrecognized in educational settings, and rarely heard in mainstream media (Eades, 2013; Malcolm, 2018).

AbE is characterized by a potentially somewhat different sound system to MAE, as well as differences in other aspects of language such as grammar, semantics, lexicon, and pragmatics (Butcher, 2008; Fletcher & Butcher, 2014). It ranges on a continuum from sounding very similar to standard AusE, to diverging markedly. Studies analyzing the phonetics of AbE in Australia are relatively few and tend to focus on L2 English speakers, in which it can be expected that features from traditional languages will have an influence (i.e., Butcher, 2008). In this current study we focus on L1 speakers, in a region in which the local traditional language (Gunditjmara) has not been spoken for many years. It is reported that there were only a few remaining speakers alive in the 1880s (Blake, 2003, p. 1), although the language is now in revival mode (Couzens et al., 2020). We do not expect, nor suggest, that phonetic differences in the varieties are related to other languages, as is seen for L2 AbE speakers, rather they are likely to be sociophonetic differences because speakers are using exactly the same phonological systems but with phonetic differences (i.e., Loakes et al., 2016 for vowels; Loakes et al., 2022 for consonants).

While some descriptive work has been carried out on the consonant and vowel systems of L2 AbE (Butcher, 2008, 2014; Mailhammer et al., 2020), very little attention has been paid to fine-grained phonetic factors, and none to perception. However, there is some, albeit very limited, acoustic work on the vowel systems of (L2) AbE, which is relevant to the current study. For both L1 and L2 speakers, it has been shown that vowels are phonetically closer in a more compressed F1/F2 vowel space than for the standard or MAE (Butcher & Anderson, 2008; Loakes et al., 2016). Evidence presented by Loakes et al. (2016) indicates that the Aboriginal L1 English cohort are not participating in all sound changes occurring for younger MAE speakers, in particular a pull chain lowering shift (see, for example, Cox & Palethorpe, 2008), which we discuss in more detail shortly. However, Loakes et al. (2016) showed that the AbE group are participating in the /el/->/æl/ merger.

### 1.2 MAE

Turning now to mainstream (or “standard”) AusE, this is the variety spoken by a large majority of Australians and is the institutional standard (Cox & Fletcher, 2017). The phonemic system of AusE is the same as Standard Southern British English, but the phonetic detail is different, particularly with regard to vowel quality (e.g., Clothier, 2019; Cox & Fletcher, 2017). While the current study focuses on regional variability in perception behavior, it is important for us to first review what we know to date about vowel production in AusE, to have an understanding of what speaker variability is present in the communities before we can understand listener reactions.

We list here some observations about MAE vowels which highlight the variability in vowels in this variety, which need to be kept in mind for interpreting the results of the listening experiment reported below. First, it has often been noted, particularly in older sources (e.g., Wells, 1982 but also Burridge (2008, p. 293)), that AusE short front vowels /ɪ e æ/ are raised such that *bag* and *beg* pronounced by some speakers of AusE may sound like *beg* and *big* to American and British listeners. This process of raising has, however, been subject to an ongoing long-term process of reversal, leading to general short front vowel lowering. This phenomenon has become increasingly evident since the 1990s, particularly among younger speakers (Cox & Palethorpe, 2008). The geographical distribution of this lowering is however still uncertain—it is known to be present in at least some major urban centers for which data have been analyzed (e.g., Cox, 1999; Cox & Palethorpe, 2008, 2019; Elvin et al., 2016), but it seems, based on our experience, much less evident in regional and rural areas of Australia where a more traditional and broader Australian accent (to which the older process of raising is linked) may be more commonly maintained. This potential patterning linked to regional/rural Australia needs to be borne in mind when we consider our perceptual data.

General short vowel lowering has also been identified as facilitating the prelateral process of /e/ merger with /æ/, reinforced if not triggered by coarticulatory effects of quite extreme lateral velarization in this variety (Loakes et al., 2017); coda laterals in MAE have been shown to be especially dark when compared with other Englishes, and other languages, for which data are available (Clothier, 2019; Schmidt et al., 2021). Second, AusE is often characterized by elevated levels of vowel nasalization, often but not solely triggered by adjacent nasal consonants (cf. Hajek, 1997; Cox & Fletcher, 2017; Cox & Palethorpe, 2014a, 2014b). In this study we look at the effect of a nasal *prior* to the vowel in nasal-vowel-consonant contexts, other AusE studies have analyzed listener reactions to prenasal /e/-/æ/ (e.g., Cox & Palethorpe 2014a, 2014b).

### 1.3 Regional variation

On to the topic of regional variability, while this is certainly very limited in AusE when compared with Englishes spoken elsewhere (see, for example, the discussion in Trudgill, 2004, p. 21; also Cox, 2006, p. 5), there are increasing indications that regional variation in the vowel system nevertheless exists to some extent. The most recent and comprehensive study on regional variability in AusE as a whole was carried out by Cox and Palethorpe (2019), who compared the vowel production of a large sample of speakers of MAE spoken across four major cities, and showed only a small number of variables were consistently and significantly different across locations; the main observations pertained to vowels and/or cities not under investigation in the current study.

Compared with the topic of regional variation in speech production which has been widely studied, correspondingly little work has been carried out into the way that listeners from different regions, who may have somewhat different production systems, cope with stimuli in perception; that is, how does regional variation play out in perception? While this has not been extensively researched, there are some studies addressing this topic. For example, Janson (1983) looked at regional variation in the perception of vowels in Swedish, Brunellière et al. (2009) carried out an event-related potential (ERP) study to analyze regional variability in vowel phoneme perception in French, and Pinget et al. (2017, 2019) focused on regional variability in voicing of the labiodental fricatives and bilabial stops in Dutch. All of these studies showed that listeners reacted differently to stimuli, depending on what they had been exposed to in their communities. The studies by Pinget et al. (2017, 2019) are important because, like the current study, they look at listener groups from multiple regions to determine regional variation. Crucially, they find that the relationship between production and perception changes as sound changes progress. With respect to the fricative change, they note that the five regions analyzed represent different stages of the sound change (Pinget et al., 2017), and in the obstruent experiment they show that a change begins in perception and completes in production, but perception “lags” (Pinget et al., 2019, p. 21) when the change is nearly complete.

Before reporting on other studies which have a similar design to our own (investigating how individuals from different locations respond to vowel categorization tasks), it is important to consider other work on English which acknowledges differing linguistic behavior in perception across dialects. While not having exactly the same design as our experiment, there are some studies (particularly on American English) which also report regional variation in perception of neighboring vowels, similar to our focus. For example, Kendall and Fridland (2012) compared the way that individuals in the United States categorized the mid-front vowels /e/-/ɛ/ in American English. Similar to the study we will describe for AusE, listeners from four locations (two northern, two southern) took part in a vowel identification study. In two of the locations, the Southern Vowel Shift was present (where crucially /e/-/ɛ/ tend to be merged spectrally, but a distinction may be maintained in length). Kendall and Fridland used 7-step continua from /e/-/ɛ/ in different phonetic contexts (specifically *bait-bet* and *date-debt*) to determine how listeners from the different locations treat variation. They showed that listeners from the four locations tended to agree on the endpoints of the continua, but with differing overall patterns depending on phonetic context. Importantly, they show that the two groups of “southerners” form a unique community, which is significantly more likely to hear /e/ than listeners from the other (unmerged) locations. Other researchers have also found perceptual effects linked to how much variability speakers are exposed to (e.g., Clopper, 2014; Clopper & Pisoni, 2004, 2006).

Research on perceptual categorization of AusE vowels is relatively limited. Mannell (2004) focused on diachronic differences in perception of AusE vowels, and this is especially relevant for the current study. Mannell examined Sydney listeners’ perceptual categorization of AusE vowels in /hVd/ contexts, across two time periods. Using both long and short vowels (monophthongs only), Mannell used synthetic stimuli which differed in F1/F2, and asked people to classify the vowels according to what they considered to be the closest AusE phoneme. Drawing on data collected in 1988 and 2004, Mannell produced perceptual contour maps which represented the frequencies at which people tended to classify individual vowel phonemes, as well as areas of overlap. Crucially, Mannell showed that for certain contrasts the perceptual boundaries had shifted over time, between the front vowels /ɪ/-/e/, /e/-/æ/ and the low/back vowels /æ/-/ʌ/ and /ɒ/-/ʊ/. Of particular interest to the current study is listeners’ reactions to the front vowels. Mannell (2004, p. 226) finds that in the time between the 1988 and 2004 sample, the perceptual boundary between /ɪ/-/e/ had become higher, while for /e/-/æ/ it has become lower. He attributes the shifted perceptual boundaries to accompanying diachronic production changes for those vowels, reported by Cox (1998, 1999), and also by Cox and Palethorpe (2008) and discussed in more detail further below.

With regard to perception of the /el/-/æl/ merger, we have previously carried out a smaller-scale study focusing on participants’ reactions to /el/-/æl/ and /et/-/æt/ stimuli in one location (Loakes, Clothier, et al., 2014, focusing on Warrnambool) and we have also carried out a more comprehensive analysis of vowel stimuli for the same contrasts across two locations (Warrnambool and Albury-Wodonga, Loakes, Hajek, et al., 2014). In the first study (Loakes, Clothier, et al., 2014), we provided evidence for the /el/->/æl/ merger in perception in MAE, and showed it was in progress rather than complete. We found that some listeners (mainly, but not solely, relatively young people) merged, while others (mainly older) did not. Focusing on the first time that listeners heard particular stimuli, rather than the repeated exposure analyzed in the current experiment, we found that listeners who merged in production tended to respond at random to /el/-/æl/ stimuli in perception, but this was not the case for the /et/-/æt/ contrast which was kept clearly distinct. The listeners who merged perceptual stimuli also tended to merge /el/-/æl/ in production, but again there was not a direct link between production and perception behavior. This finding is not unusual in merger situations: as mentioned earlier there can be a misalignment in production and perception when sound changes are in progress. In Loakes, Clothier, et al. (2014) it was considered that different input from different talkers, along with the sometimes ambiguous stimuli received in input, could have an ongoing effect on the ability to classify stimuli. Similar kinds of results have been discussed for the merger of /el/-/æl/ in New Zealand English by Hay et al. (2013), although they also note that “. . .[el] phonetic tokens are . . . not as ambiguous as the [al] phonetic tokens, which could more plausibly represent either category” (p. 251). They account for this through the hybrid model of speech production and perception, whereby people’s differential behavior in production and perception is influenced by episodic memories of other people’s speech (following e.g., Pierrehumbert, 2006; see also Sumner & Samuel, 2009). In this view, speech perception has also been called more “malleable” than production (Beddor et al., 2018).

This “misalignment” in production and perception is also implicated in language change more broadly. This is also at the heart, for instance, of the interactive-phonetic (IP) sound change model outlined by Harrington et al. (2018). In this model of production and perception, they illustrate that listener’s decision boundaries can shift and skew depending on interactions between speakers (see esp. Harrington et al., 2018, pp. 3–4). Along with all the factors involved in sound change and discussed so far, the IP model also includes a focus on the role of (certain) individuals as responsible for the initiation and propagation of sound change (Harrington et al., 2018). This important role of individuals is also taken up by other researchers, for example, Baker et al. (2011, p. 347), who observed that “sound change is predicted to be biased toward phonetic effects that exhibit interspeaker variability.” In a study focusing on production and perception of vowels in Dutch, Voeten (2021) also highlights the role of individuals in adopting changes which eventually propagate into community-based change.

The studies described in this section are a good reminder that not everyone in a speech community will behave in a linguistically similar way to the same stimulus, because of their experiences listening to different speakers in their communities.

## 2 Aims

The primary aim of this work is to investigate which sociophonetic factors influence perception of AusE, to understand the judgments listeners make in categorizing vowels which are undergoing change. This will ultimately help us gain insights into how phonetic processing works in the context of language change. In addressing these issues, we explore a range of variables.

### 2.1 Regional variation

We address how groups of Australian listeners across four locations categorize and interpret vowel stimuli. Specifically, we focus our attention on vowel categories which are highly variable due to diachronic change (in the short vowel system over time) as well as the /el/-/æl/ merger in progress. Following the results seen in the United States by Kendall and Fridland (2012), we predict that listeners will not act the same as one another precisely due to acoustic variability in the vowels under analysis, and due to levels of exposure to merged variants in their communities.

### 2.2 Social variation

We consider the way that two socially different groups act when it comes to perception behavior. The AbE-speaking group which we focus on has traditionally tended to have separate social interactions from the non-Aboriginal people in the same community, and certainly have different vowel targets (e.g., Loakes et al., 2016). We predict differences in their interpretation of the vowel stimuli as a result.

### 2.3 Age

As mentioned earlier, diachronic variation has been rapid in AusE and this has meant that there is variation in vowel production across generations. We predict similar variability in perception behavior because of this.

Specific research questions for this study are:

1. What are the geographical limits of the /el/-/æl/ merger in Victoria?
2. How do listeners from different locations in southeast Australia categorize vowels generally – do listeners from different locations have different crossovers (i.e., is there regional variation in perception)?
3. How do L1 AbE listeners categorize MAE vowels (i.e., is there varietal difference in perception)?
4. What role does listener age have on categorization behavior for AusE vowels, and for “merger behavior”?

## 3 Data and methods

### 3.1 Listeners and listener groups

The experiment reported here is also a first attempt to assess variation in speech perception across multiple AusE-speaking communities. The listeners are from two major social/accent groups: L1 AbE speaking, and MAE (MAE). The AbE group is from one location (WN), while the MAE group are from four locations—all within southeast Australia. Further details are given below about these locations.

A total of 81 listeners in total took part in the study. This is further broken down in Table 1.

**Table 1.** Participant Demographic Distribution (Site, Variety, Mean Age, Age Range).

Site	Variety	M	F	Age group			
				Younger (*n*)	Older (*n*)	Mean age	Age range
AW (Albury-Wodonga)	MAE	4	11	7	8	44	22–61
MI (Mildura)	MAE	4	9	8	5	40	18–72
ML (Melbourne)	MAE	7	9	5	11	43	20–78
WN (Warrnambool)	AbE	10	12	15	7	33	18–64
	MAE	8	7	8	7	42	19–73
Totals		33	48			40.4	18–78

*Note.* MAE: Mainstream Australian English; AbE: Aboriginal English.

The study includes 33 males and 48 female listeners. For the accent groups, this equates to 59 MAE listeners (23 male, 36 female) and 22L1 AbE listeners (10 male, 12 female). Of the 59 MAE listeners, 15 are from WN, 15 from AW, 13 from MI, and 16 from ML. Due to a natural split in the data, we divided participants into “older” (above 40) and “younger” (under 40).

The AbE listeners are on average younger than the MAE listeners, and age is accounted for in the analysis.

**Figure 1.**
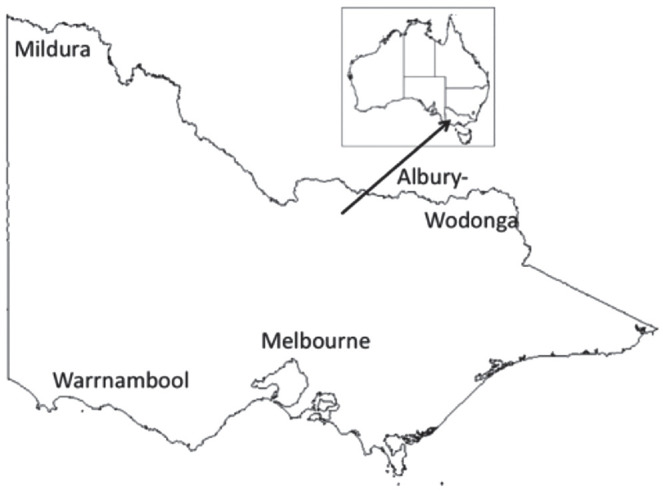
Map of Australia (small), with the state of Victoria (large). The four sites are labeled in their approximate locations.

Participants were visited in their communities, either at home or in a public area, depending on their preference. Three of the sites in this study—Albury-Wodonga, Mildura and Warrnambool—are “regional” towns on the northeast, northwest, and southwest periphery, respectively, of the state of Victoria. Melbourne, by contrast, is a large capital city located in the south-central region of the state. Mildura and Albury-Wodonga are “northern” towns, while Melbourne and Warrnambool are “southern.” Locations can be seen in Figure 1.

While relatively small within the Australian geographical context, Victoria has the second largest number of inhabitants by state with around 6 million people, (Australian Bureau of Statistics [ABS], 2016). Australia has in total 2.8% people who identify as Aboriginal, with differing numbers of Aboriginal people in communities, depending on the location. Victoria has the lowest numbers of people (0.8%) who identify as Aboriginal compared with any other state in Australia (ABS, 2016).

Looking more closely at the map in Figure 1 and the relative locations of the communities included in this study, it is apparent that Mildura’s geographical location intersects with three state borders. It is located within Victoria, but is particularly close to the state border of New South Wales (NSW). The Murray River, running at the northern edge of the township, represents the NSW/Victorian border, while South Australia is approximately 110 km away (around a 1-hour drive). In our study, this is the most remote location in terms of distance from capital cities. Mildura is approximately 540 km (335 miles) from Melbourne, but only 390 km (242 miles) from the South Australian capital of Adelaide, to which residents of Mildura often prefer to travel, rather than to Melbourne, for important services. It is an arid region (which is now a fruit and winegrowing region due to river irrigation) and is sometimes referred to as a “desert oasis” (see, for example, Ryder, 2011).

Warrnambool is a coastal town in southwest Victoria. It has a population of just over 30,000 people, of whom approximately 500 are Aboriginal (Office of Aboriginal Affairs Victoria [OAAV], 2014). Warrnambool is 256 km (159 miles) from the capital Mildura and is close to the scenic “Great Ocean Road,” which is a popular destination for tourists from Melbourne and elsewhere.

Albury-Wodonga on the northeast side of is effectively two connected towns which are divided by the NSW–Victorian border, and these are often dubbed as “twin cities” (see, for example, Wright, 2010). In total, the population is said to be approximately 90,000, with around 39,000 in Wodonga on the Victorian side of the border, and the rest in Albury. Participants in this study live on both sides of the border (and cross freely and regularly). While Albury-Wodonga is much closer to Melbourne than to Sydney, the capital of NSW, some residents especially of Albury-Wodonga, prefer to travel to Sydney for important services.

Melbourne, by contrast to these relatively small towns, is the second largest city in Australia with approximately 4.5 million inhabitants (ABS, 2016). Melbourne is also extremely multicultural, with 40% of people born outside the country (ABS, 2016). By contrast, the other locations in this study have fewer people born overseas; 25.9% in Warrnambool, 20.6% in MI, and 17.9% in Albury-Wodonga (ABS, 2016).

All our participants were born in, and have lived in either Warrnambool, Melbourne, Mildura, and Albury-Wodonga for most or all of their lives. We specifically planned the study in this way because we wished to examine the geographical limits of the merger (and could not reliably do this with highly mobile participants). Previous work has shown that participant mobility (and thus exposure to variability) impacts on the ways that listeners react when exposed to acoustic stimuli (e.g., Clopper, 2014; Clopper & Pisoni, 2006; Loakes et al., 2019; Sumner & Samuel, 2009). We know that the listeners will have marked differences in the variation they receive in input; Melbourne, by world standards, has an especially diverse population, while the regional locations have far less.

### 3.2 Experimental task

The task is presented on an iPad using a custom app designed specifically for the task (see Loakes, Clothier, et al., 2014). Individual words were played to listeners via headphones (Shure SRH840 Reference Studio Headphones), and items were also presented orthographically on “buttons” on the screen. Of the two options presented, listeners made a choice by pressing which of the two items they had heard before moving on to the next item. The items involved 7-step continua, which we describe in more detail below. The target words we use in this study (and the resulting continua) are designed to focus on categorization of front vowels in various contexts, broadly /hVt/, /hVl/ and /mVl/. The contrasts are shown in Table 2.

**Table 2.** The Six Contrasts Analyzed in This Study.

	/i-e/	/e-æ/
/hVt/	*hit-het*	*het-hat*
/hVl/	*hill-hell*	hell-Hal*
/mVl/	*mill-Mel*	Mel-Mal*

*Note.* “Merger” conditions are labeled with *.

Listeners were timed for each stimulus, and could not replay the item, go backwards in the experiment, or change their mind once a decision was made. Every item was presented four times, with the orthographic representation shown twice on the left side of the screen, and twice on the right.

The target words that we presented to listeners involved a mix of low- and high-frequency words, and included names. Lexical frequency has been shown to be important for understanding merger behavior in many studies (e.g., Hay et al., 2006; Kang et al., 2015), although a recent study on this particular merger has shown that lexical frequency is not a factor in promoting speaker behavior among an older cohort (Schmidt et al., 2021). Nevertheless, lexical frequency of the items has been assessed to help with interpretation of results. The only lexical frequency database for AusE, ICE-AUS, is small in comparison with other available English corpora, and so frequencies from SUBTLEX-UK (van Heuven et al., 2014) are reported in Table 3, with some small comment underneath regarding AusE (from ICE-AUS). Words are ranked from highest to lowest frequency. Names feature in both databases, so rankings are available for names as well. Given that naming practices and trends change across generations, trends for naming in Australia have been analyzed and are reported further in Table 4.

**Table 3.** Relative Lexical Frequency Reported in Raw Figures.

SUBTLEX	
hit	41,043
hell	15,495
hat	11,557
hill	10,370
mill	3,739
Mel	2,491
Hal	423
Mal	383
het	90

**Table 4.** Popularity Ranking of Names Used in Experiment (in Australia).

	1944	1984	2000	2013
Hal	84	722	442	495
Mel	0	56	224	818
Mal	15	413	495	1,738

Comparing these frequencies with the far smaller ICE-AUS, the ordering is almost the same, with *hit* being most frequent (but only 155 entries total), and *hill, hell, and hat* having relatively high rankings (but in a different order), *mill* occurring in the middle, and *het* plus the names *Hal, Mal*, and *Mel* having the lowest frequencies (but in a different order, and *het* and *Hal* having no entries at all). Naming data is drawn from the most popular male and female baby names in Australia from 1944 to 2013 (South Australian Government, 2020); data outside this period are not available. We show rankings of the names used in the database in 1944, 1984, 2000, and 2013 to give a sense of frequency of use over the time periods. A ranking of 1 would mean the name is the most common given to babies in a particular year; higher rankings mean the name is less popular and therefore has less common usage overall.

The table shows that the male names *Hal* (short for *Harold*) and *Mal* (short for *Malcolm*) showed a dramatic drop in usage across the span for which data are available, highlighting that these names are more common for older rather than younger people, and therefore likely more frequent among the older listeners in our study. As also mentioned above, *Hal* was not present in the ICE-AUS database at all. The female name *Mel* (short for *Melanie* and *Melissa*) did not occur at all in the 1944 database, had a relatively high frequency of use in 1984 and 2000, and has become relatively low frequency by 2013. This indicates that *Mel* should be a relatively frequent item among our younger listeners in particular. We return to the role of lexical and naming frequencies later, in considering some of the results.

**Figure 2.**
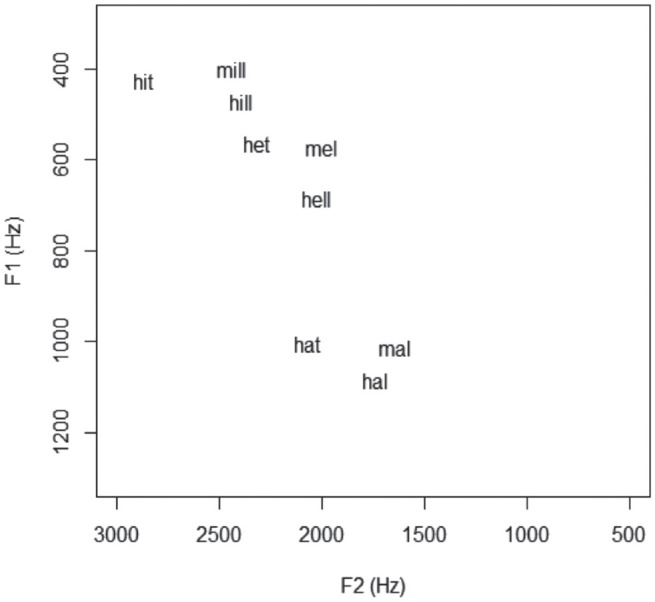
F1F2 vowel space showing the F1/F2 relationship between contrasts presented to participants.

As for the vowel data, each vowel continuum began and ended with a single real speech token produced by a 40-year-old native female AusE speaker from a capital city (Brisbane in Queensland), where merger is not reported. At the time of the recording she had lived in Melbourne for 5 years. The use of one voice, rather than multiple talkers, was important for the goals of this study—other studies have shown that multiple talkers cause accuracy issues and timing delays in processing (see Clopper (2014, p. 70) and references therein). Crucially, the speaker maintained the /e/–/æ/ contrast in all contexts, as seen in Figure 2.

Between each end of the continua were five equidistant tokens (in raw acoustic terms) manipulated for F1, F2, and F3. To create the stimuli, 7-step continua were created using the Akustyk (Plichta & Preston, 2004) vowel synthesis module in Praat (Boersma & Weenink, 2019). Duration was not altered. There was a total of four presentations of each token and these were presented at random, resulting in a total of 196 trials per listener. Our procedure, including stimulus manipulation and presentation, followed closely that used by Harrington et al. (2008) and Kleber et al. (2011), albeit with fewer step points in the continua in our study. It also uses very similar methods to the study reported earlier by Kendall and Fridland (2012). For each contrast pair, formant measurements^3^ for the endpoints of the continua are listed in Table A1 in the Appendix. Each of the intervening stimuli were equidistant between these endpoints.

Figure 2 shows the stimuli plotted on an F1/F2 vowel space, to better understand of the input listeners heard when listening to the experiment—this of course does not count for the entire vowel trajectory but gives a good indication of the relative acoustic difference between the stimuli (formants are also reported in Hz in Table A1 in the Appendices). The figure shows Steps 1 and 7 of each contrast pair (the intervening steps are incremental and synthesized, as mentioned). Importantly, the same file was used for the same target word in the different contrast pairs, and for repetitions. For example, the 7th step in the *hill-hell* continuum (a naturally produced *hell*) was the same file as used in step 1 of the *hell-Hal* continuum. Likewise, every occurrence of that particular *hell* (whether Step 7 in *hill-hell* or Step 1 in *hell-Hal* was exactly the same).

Some observations which are important for understanding the results are as follows:

- The F1 distance between the KIT and DRESS vowels is smaller than the F1 distance between the DRESS and TRAP vowels (this is typical of the “new” and very open AusE /æ/ vowel, as discussed earlier);
- In F1, the /hVl/ condition is always phonetically lower and more central across each of the KIT-DRESS-TRAP contrasts, which is unsurprising given the known coarticulatory effects of the post-vocalic lateral;
- This speaker keeps *hell-Hal* distinct (i.e., she does not merge in production). However, there is more phonetic distance between the “control” and prelateral condition for the DRESS vowel, than for other contrasts. That is, when comparing *hit-hill, het-hell*, and *hat-Hal* the speaker used slightly more lowering for *hell* than for *hill* and *Hal.*
- In the /mVl/ contexts, the onset nasal causes the speaker to produce a systematically phonetically higher vowel than in /hVl/—a predictable raising effect known to occur in AusE;
- In F2, the “control” condition /hVt/ is always more front than the /hVl/ and /mVl/ counterparts; in other words, the lateral causes retraction. This is not surprising, as we know this coarticulatory effect occurs in many Englishes.

### 3.3 Statistical analysis

We built mixed effects binomial logistic regression models using the glmer function in lme4 (Bates et al., 2015) using the RStudio IDE (RStudio Team, 2020) for R (R Core Team, 2020). For each model, we began with a baseline intercept-only model, and proceeded testing the explanatory contribution of different fixed factors (continuum step, site [where relevant], age group, gender, listener variety [where relevant], and interactions between these factors), using a stepwise step-up procedure. The final model in each case is the minimal adequate model, and we report the results of likelihood ratio tests comparing these minimal adequate models with their respective baseline intercept-only model. In addition to the fixed intercept, the baseline and more complex models had by-listener random intercepts.^4^ Treatment coding was used for all categorical predictors and polynomial coding was used for the ordinal predictor step. For each model, the outcome variable was the identified vowel. In addition, for each contrast modeled, the word appearing at the left edge of the respective data plot was treated as 1 and the right edge 0 (i.e., for *hell-Hal, hell* was treated as 1 and *Hal* was treated as 0). In addition:

- When modeling age group: the older group was treated as the reference level;
- When modeling site: Albury-Wodonga was treated as the reference level because it has been shown to be more representative of general MAE, wherein speakers do not participate in the merger under study (Loakes, Hajek, et al., 2014);
- When modeling by variety: AbE was treated as the reference level because speakers of AbE have been shown to have less participation in general vowel changes occurring in AusE (Loakes et al., 2016), specifically the pull chain lowering shift (Cox & Palethorpe, 2008) which we suggest is more broadly linked to the merger discussed in this paper.
- Binary coded gender did not prove a significant factor in any of the analyses; however, in the construction of models that led to its elimination from the final minimal adequate models, the reference level was “man” (however, it should be noted that choice of reference level does not affect the overall fit of a model).

Due to the number of comparisons we have constructed by using separate models per continuum, we have calculated a Bonferroni-corrected alpha level for “statistical significance,” which derived .008. Any mention of statistical significance is with respect to this alpha level, and any *p*-values that are significant with respect to this alpha level are marked with an asterisk. Summary tables can be found in the Appendices (A2-A16).

## 4 Results

This section details results of the listening experiment across the five communities (4 locations/two speaker groups). Participant responses are plotted, and for each contrast there is a site-wise comparison (across the four MAE listener groups) and then comparison per variety (Aboriginal versus non-Aboriginal within WN). Response data are further segmented by age group. After each plot, the results of the statistical modeling are presented.

### 4.1 Participant responses (categorization behavior)

#### 4.1.1 hell-Hal: predicted merger condition

**Figure 3.**
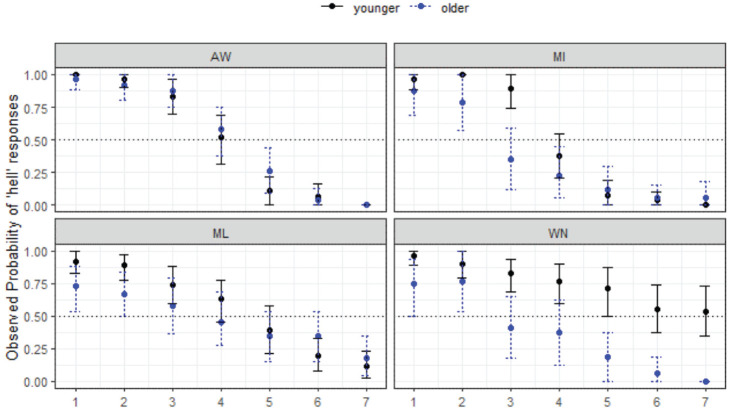
Responses for hell-Hal continuum by site and age group (MAE speakers).

Figure 3 shows the responses for the four MAE communities. For each subfigure, stimulus step is shown on the *x*-axis and the proportion of responses (as observed probabilities or proportions of responses) on the *y*-axis. In this case, where the responses are for *hell-Hal*, the naturally produced *hell* token is Step 1 and the naturally produced *Hal* is Step 7 (with Steps 2–6 equidistant in between, as described earlier). Throughout this section, the plots always show listener reactions to the first-mentioned word, in this case *hell*. If at Step 1 the point reaches 1, this means that all listeners in the given location/age group chose *hell* for all trials. If at Step 7 the point reaches 0, then all listeners chose *Hal* for all trials. If the point does not reach 0 by Step 7, this means that some listeners were still selecting *hell* and we infer this to be indicative of merger. The “crossover” is the point at which the majority of listeners switch categories between the two options, either above or below the 0.5 line that runs horizontally through the middle of the figure. Responses also show age differences in each community (younger shown in black solid line, older in blue dotted line).

The results show that patterns of listener behavior are different among the four communities, and that, overall, younger listeners tend to show *less* merger behavior than older listeners, although in WN (bottom right) the pattern is reversed. The younger listeners in this community are answering randomly at this end of the continuum, which is a clear case of merger, because the /æl/ stimuli are ambiguous for them and these younger listeners never cross over to *Hal*. The older WN listeners have some merger behavior at Step 1, but then declining *hell* responses along the continuum (i.e., overall, there is a contrast in perception for this group). In AW (top-left), the results show no merger behavior at all for either age group, with almost all listeners choosing *hell* with 100% reliability when Step 1 *hell* was heard, with just one *Hal* response. Results are similar for the MI listeners (top-right). The results here show the two northern sites patterning very similarly, with effectively no merger behavior overall for most listeners, but some ambiguity around Steps 1 and 7 for the older MI listeners among the older group only.

For the southern sites, ML and WN (bottom-left and bottom-right, respectively), the responses are more varied. For ML, there is an overall pattern of gradually disfavoured *hell* responses from Steps 1 to 7, as opposed to the sharp decline seen in AW and MI. There is some clearer evidence of merger behavior for these southern sites, which plays out differently in each of the locations (and, as mentioned for WN, across age groups). In ML it is clear that there is some merger behavior at each end of the continuum (more so for older speakers), with some speakers choosing Step 1 for Step 7, and vice versa. For WN this merger behavior is more marked. The older MAE listeners show some difficulty categorizing *hell* (some choose *Hal*), but are clear on their responses at Step 7. However, the younger MAE speakers in this community have an unexpected tendency to choose *hell* and answer randomly at Step 7. Both the younger and older WN groups here show merger behavior, but are affected in different directions. The effect of the merger is much more marked for the young WN group.

**Figure 4.**
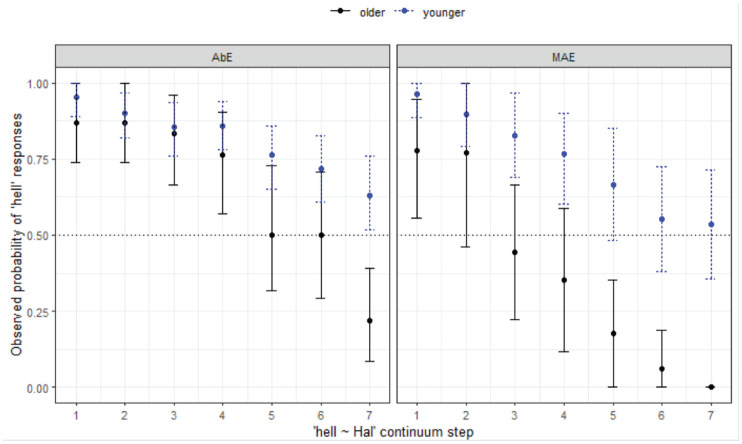
Responses for hell-Hal continuum by variety and age group (WN).

Statistical modeling shows the final minimal adequate model performed significantly better than the baseline model, χ^2^ (15) = 783, *p* < .001, and has good fit (C = .927; Somer’s D_xy_ = .853). This model confirms site-wise differences, wherein compared with AW, ML (*z* = −4.80, *p* *<* .001*) and WN (*z* = −4.25, *p* < .001*) are less likely to respond with *hell* overall, and reveals a significant 3-way interaction between continuum step, site, and age group, whereby the older listeners in ML (*p* = 2.95, *p* = .003*) are more likely to respond with *hell* as step increases, but this is not the case for older listeners in WN (*z* = −1.39, *p* = .165), where the reverse is plainly true per Figure 4. age group is best modeled as an interactive term (rather than as a main fixed effect only), χ^2^ (7) = 29.298, *p* < .001.

Turning now to a comparison of the groups in WN with different backgrounds in terms of language variety—MAE and AbE—there is a distinct pattern of responses as seen in Figure 4.

Figure 4 shows merger behavior in both groups, and both have within-group differences depending on listener age, wherein younger speakers are more likely to respond with *hell* as step increases (*z* = 2.97, *p* = .003*), but the age differences appear more marked for the non-Aboriginal group (while not statistically significant based on our modeling). In WN, the younger listeners, regardless of social group, have more merger behavior than the older listeners across the board, and older Aboriginal listeners have some ambiguity in perception of *Hal* which is not present for the older mainstream listeners. Overall, non-Aboriginal listeners appear less likely to respond with *hell* across the continuum, although this is not a statistically significant finding (*z* = −2.33, *p* = .020).

The final minimal adequate model reveals a significant 2-way interaction between continuum step, age group, and listener variety. This model performed significantly better than the baseline model, χ^2^ (4) = 171, *p* < .000, and has good fit (C = .866; Somer’s D_xy_ = .731). In other words, the Aboriginal listeners are responding significantly differently from the MAE listeners for this contrast, although the differences are not as marked as the site-wise variability seen for the MAE listeners.

#### 4.1.2 Het-hat: control condition

**Figure 5.**
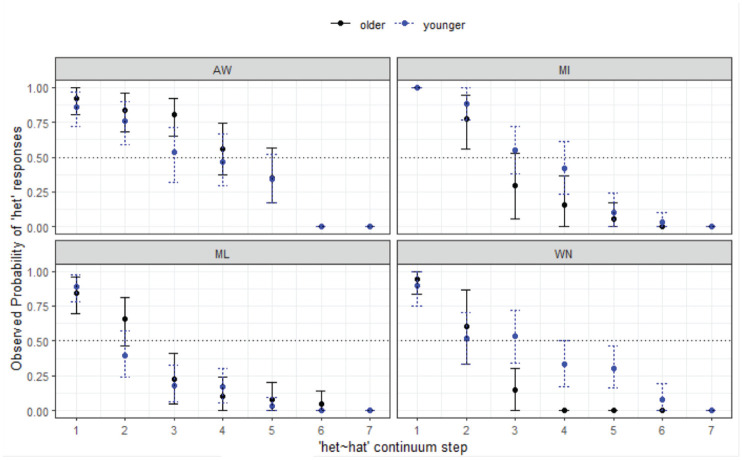
Responses curves for het-hat continuum by site and age.

Turning now to the *het-hat* condition, which was designed to be a “control”; since merger has not been reported in the literature for this context, results indicate that there are unexpected differences across the sites in how the contrast is categorized from the outset. Figure 5 shows how categorization behavior plays out across the groups and communities.

The final minimal adequate model performed significantly better than the baseline model, χ^2^ (15) = 954, *p* < .001, and has good fit (C = .964; Somer’s D_xy_ = .928). The model confirms there are site-wise differences and reveal a significant 3-way interaction between step, site, and age group. Regarding the groups as a whole, listeners in ML are more likely to respond with *hat* across the continuum (*z* = −3.01, *p* = .003*). As seen in Figure 5, there is good agreement of the endpoints in the *het-hat* contrast by the AW group, with the older listeners having a later crossover than the younger listeners (the younger listeners switch to *hat* more quickly). In MI and WN, there is a trend for the younger listeners to have later crossovers, but the response patterns are more variable for these listeners (and age differences are more marked in WN). Overall, there is a trend for younger listeners in MI (*z* = 2.33, *p* = .020) and ML (*z* = 2.04, *p* = .042) to respond with *het*. In ML, this likelihood decreases with increases in step, (*z* = −2.40, *p* = .016)

The ML listeners are especially interesting, patterning differently compared with the other regions. They show fewer differences in how the younger and older listeners respond, as well as less agreement at the *het* end of the continuum. These responses for ML suggest that the earlier described pattern of vowel lowering, confirmed in urban areas, is driving differing behavior at the *het* end of the continuum for these urban listeners only. The control, by chance, thus happens to be a good one for the rural listeners, and especially the AW listeners, but less so for the urban listeners.

**Figure 6.**
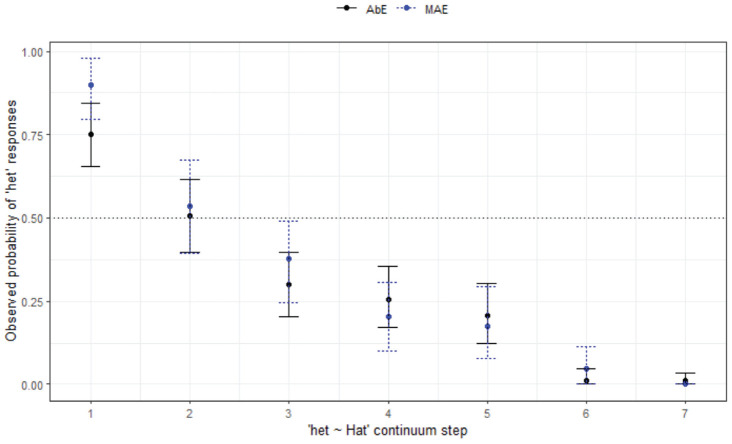
Responses for het-hat continuum by listener variety (WN).

The plot for the AbE listener responses to *het-hat* (Figure 6) looks very similar to that for the MAE WN group.

The final minimal adequate model performed significantly better than the baseline model, χ^2^ (3) = 404, *p* < .001, and has good fit (C = .933, D_xy_ = .865). This model shows an interaction between listener variety and step (not age), with MAE listeners overall more likely to respond with *het* (*z* = 635.94, *p* < .001*), but also less likely to respond with *het* as step increases (*z* = -173.52, *p* < .001*). As Figure 6 shows, MAE listeners tended to respond slightly more with *het* than *hat* in the first three steps of the continuum compared with their AbE counterparts. As this result does not match with what we know about AbE production (having closer vowels, and therefore we should expect more *het* responses), we might infer there may be some lexical frequency differences *within* the communities, although this remains an empirical question for now, as there is no widely available AbE corpus that can be used for lexical frequency. It is also of course possible that there is simply a mismatch in production and perception for these listeners—this would not be unusual, especially in the case of merger behavior (see, for example, Harrington et al., 2018; Hay et al., 2013).

#### 4.1.3 Prelateral /ɪ-e/: hill-hell

**Figure 7.**
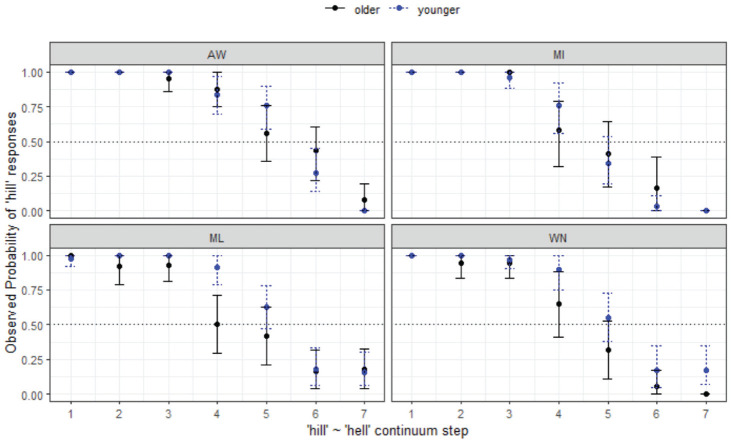
Responses for hill-hell continuum by listener site and age group.

This section focuses on the results for another control condition, this time the prelateral close-front vowels /ɪ-e/ which have not been reported to be undergoing any prelateral merger in AusE. This is also confirmed in the current study. The final minimal adequate model for this contrast performed significantly better than the baseline model, χ^2^ (1) = 1,087, *p* < .001, and has good fit (C = .971, D_xy_ = .942). This model shows that the only effect on listeners’ responses is continuum step (*p* < .001): there is no effect of site or age group. In other words, all listeners respond in effectively the same manner across age groups and location. That continuum step is the only important factor in categorization of this contrast, with only minimal age × location differences, as seen in Figure 7.

These results for *hill-hell* are especially interesting for the study overall because they show regional *similarity* for the four MAE sites, as opposed to the divergent responses seen among them when the highly variable /e-æ/ was included in the analysis.

**Figure 8.**
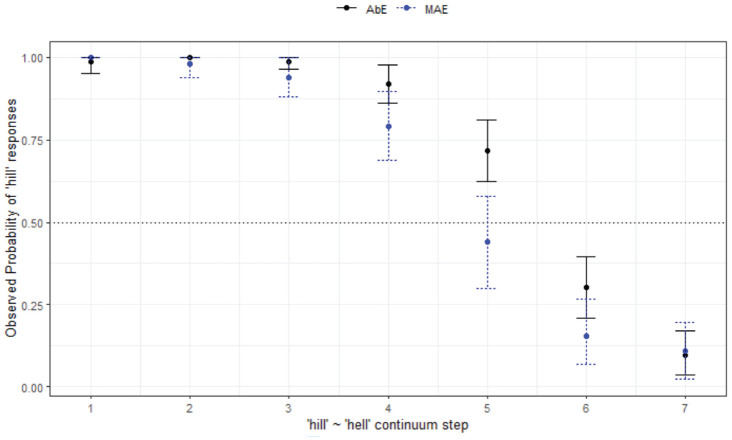
Responses for hill-hell continuum by listener variety (WN).

Considering the effect of variety, the final minimal adequate model for *hill-hell* in WN performed significantly better than the baseline model, χ^2^ (2) = 646, *p* < .001, and has good fit (C = .963, D_xy_ = .926). This model shows significant main effects of step (Figure 7) and no significant effect of variety (i.e., minimal differences between Aboriginal and non-Aboriginal listeners, as seen in Figure 8).

#### 4.1.4 hit-het

**Figure 9.**
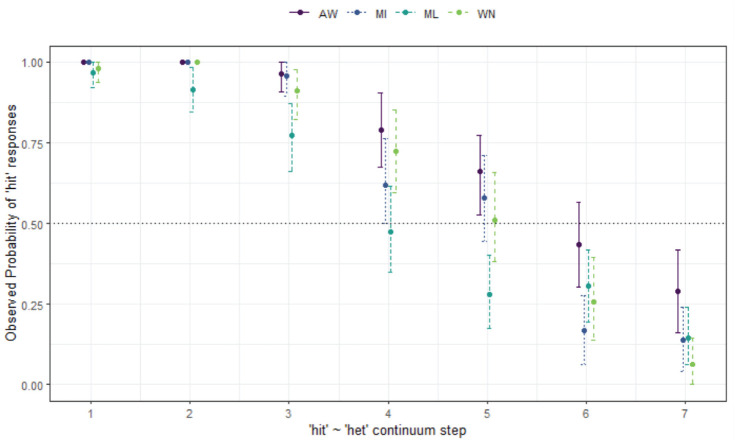
Responses for hit-het continuum by listener site.

Turning now to the non-lateral *hit-het* continuum, there is a difference in that the urban listeners react differently, which is interesting because it shows previously unnoticed regional variability in perception. Participants in ML tend to provide more *het* responses across the continuum and also cross over from *hit* to *het* at an earlier point in the continuum at Step 4, while the rural groups cross over by Step 6 (Figure 9). There are no other age or location differences, and in fact WN and MI share very similar response patterns. These patterns are supported by the statistical modeling, which only returns step, site, and an interaction between step × site as significant predictors. The final minimal adequate model performed significantly better than the baseline model, χ^2^ (7) = 944, *p* < .001, and has good fit (C = .966; Somer’s D_xy_ = .932).

Comparing responses to *het* in Figure 9 here and for the *het-hat* contrast shown in Figure 5, it appears that there is a general tendency not to choose *het.* In the contrast here, the crossover to *het* is somewhat late, while the crossover to *hat* was somewhat early, indicating that (despite some regional variability), lexical infrequency of *het* may play some role in listeners’ choices when responding.

**Figure 10.**
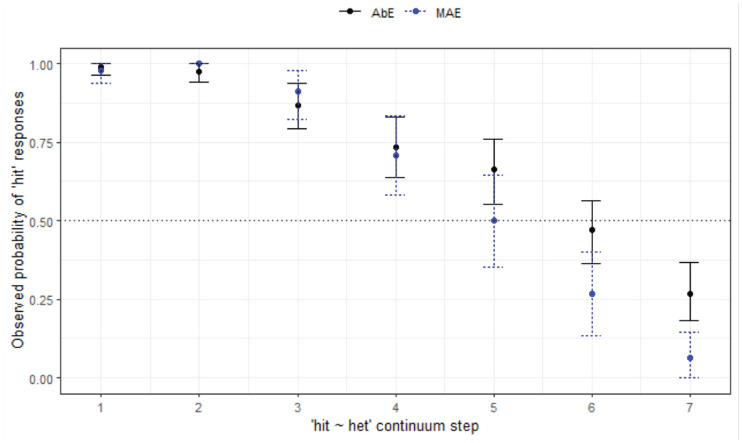
Responses for hit-het continuum by listener variety (WN).

With regard to the WN responses, Figure 10 highlights the variability observed between the varieties, with the AbE listeners responding with *hit* for longer, and not reaching 100% agreement by Step 7 (73% for AbE and 94% for MAE listeners). The MAE listeners switch to *het* sooner and with more agreement among the group, more consistently decreasing in *hit* responses across the continuum (*z* = −2.69, *p* = .007*). Similar to the discussion above for MAE listeners, this finding may well be motivated by a tendency not to choose *het* within the AbE listener group, and is also likely motivated by the fact their vowels are phonetically closer, so they hold on to *hit* for longer. The final minimal adequate model for /*hit-het*/ in WN performed significantly better than the baseline model, χ^2^ (3) = 493, *p* < .001, and has good fit (C = .953, D_xy_ = .906). This model shows an interaction between continuum step and listener variety, supporting the observations discussed above and shown in Figure 10.

#### 4.1.5 Post-nasal, prelateral environments: mill-Mel

**Figure 11.**
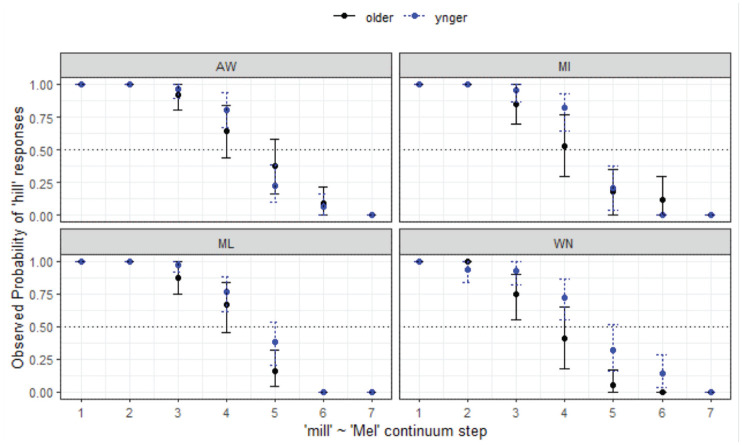
Responses for mill-Mel continuum by listener site and age.

We turn now to the post-nasal and prelateral conditions, to (indirectly) examine the effects of different types of coarticulation on AusE vowel categorization. The focus is first the /ɪ-e/ context. Figure 11 shows the *mill-Mel* continuum. The final minimal adequate model for this continuum performed significantly better than the baseline model, χ^2^ (1) = 1,365, *p* < .001, and has good fit (C = .978, D_xy_ = .956). This model shows that the only effect on listeners’ responses is continuum step (*z* = −15.63, *p* < .001): there is no effect of site or age group. This is similar to the findings for *hill-hell* shown earlier, with no difference across the four locations and age groups in their response to these stimuli. These results suggest that this prelateral /ɪ/-/e/ context is least “difficult” and differentiating of all those examined in terms of site-based variability.

**Figure 12.**
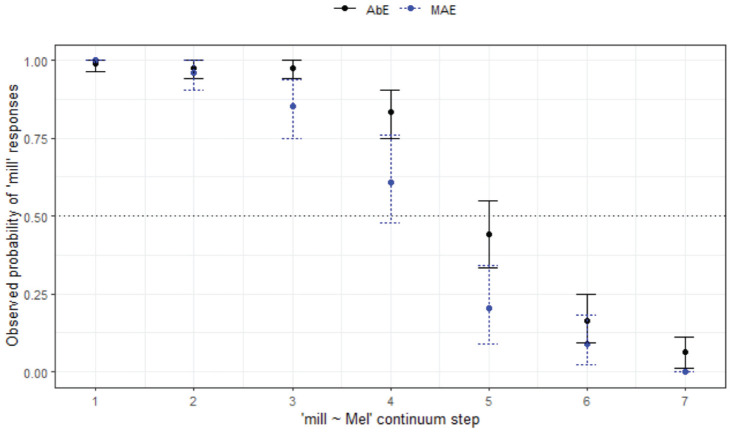
Responses for mill-Mel continuum by listener variety.

To compare the Aboriginal and non-Aboriginal listeners within WN (Figure 12), the same kind of model to those reported previously was fit to the data. The final minimal adequate model performed significantly better than the baseline model, χ^2^ (3) = 701, *p* < .001, and has good fit (C = .952, D_xy_ = .904). This model shows significant main effects of step and age whereby younger listeners are more likely to respond with *mill* across the entire continuum (*z* = 3.17, *p* = .002*), and here we also see difference by variety whereby MAE listeners were less likely to respond with *mill* across the entire continuum (*z* = −3.01, *p* = .003*).

#### 4.1.6 Post-nasal, prelateral environments: Mel-Mal

**Figure 13.**
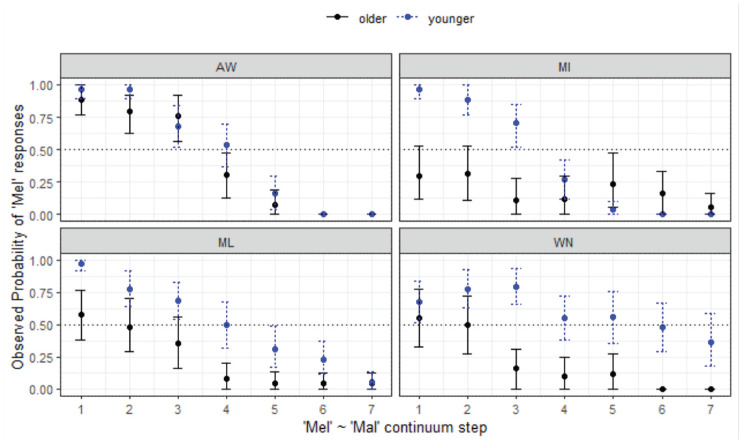
Responses for Mel-Mal continuum by site.

Of all the contrasts analyzed, the *Mel-Mal* context is expected to produce highly variable responses: it is a predicted prelateral merger context, and the preceding nasal was also expected to influence responses due to carryover coarticulatory effects. These are contradictory forces, and it appears the lateral has the strongest effect (in some communities) as seen in the patterns shown in Figure 13.

The analysis shows that all three southern sites are less likely to respond with *Mel* overall (*z* = -5.15 to −3.32, *p* < .001*). There is also a significant 3-way interaction whereby younger listeners in MI are less likely to respond with *Mel* as step increases (*z* = −3.35, *p* = .001*). Conversely, older speakers in MI tend to respond with *Mal* across the continuum, and younger speakers in WN tend to respond with *Mel*. Looking more closely at the cohorts in each of the locations, it is evident that 100% agreement is only achieved for AW listeners, and young listeners in MI—all other groups show variability in their decisions at one or both endpoints. This analysis shows that while merger behavior is present in perception for many of the listeners, it plays out in different ways, and we might predict that this could be due to the interplay between merger and lexical frequency effects.

**Figure 14.**
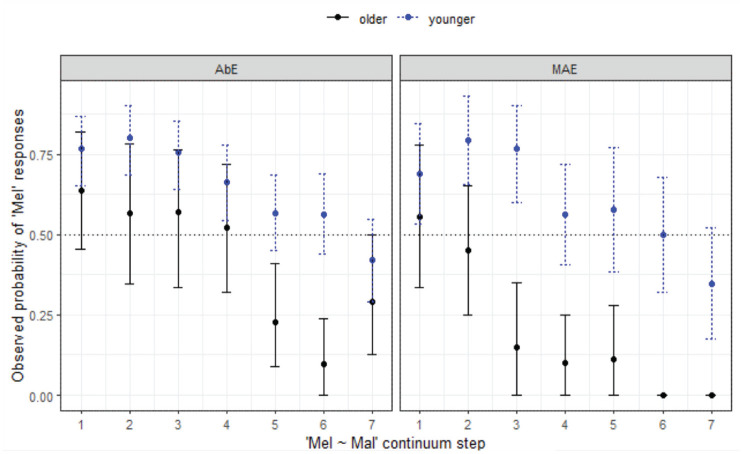
Responses for Mel-Mal continuum by listener variety (WN) and age group.

Turning now to a comparison between the Aboriginal and non-Aboriginal listeners (Figure 14), the AbE listeners perform similarly to their MAE counterparts in WN in terms of responses but age differences are stronger for the mainstream listeners.

The final minimal adequate model for these data performed significantly better than the baseline model, χ^2^ (7) = 125, *p* < .001, and has good fit (C = .841, D_xy_ = .681). The model reveals a significant 3-way interaction between step, variety, and age group, showing more substantial differences in response between age groups across the continuum in the mainstream AusE group (whereby the younger MAE listeners are more likely to respond with *Mel* even as step increases) compared with the AbE group (*z* = 2.05, *p* = .041).

Both Aboriginal and MAE listeners exhibit merger behavior in WN. Only the older MAE group reaches 100% agreement at the *Mal* end of the continuum and neither the younger or older cohort has close to 100% agreement at the *Mel* end of the continuum; that is, there is variability in whether they are hearing *Mel* or *Mal*. As can be seen in Figure 14, similar to the non-Indigenous listeners from this location, the older group tends to choose *Mal* over *Mel*, and this pattern is reversed for the younger listeners.

## 5 Discussion and conclusion

This study compared responses to a listening task with participants from four different locations (ML, WN, MI, and AW) and two community/heritage groups (Aboriginal and MAE-speaking) to determine which factors are important in categorizing AusE vowel stimuli.

Analysis of listener responses shows various factors are at play with regard to how the groups respond to the stimuli. Our modeling showed that phonetic contrast and (relatedly) continuum step were significant factors in response patterns. We also saw that location, listener age and variety spoken (mainstream and Aboriginal AusE) were additional important factors driving the patterns of responses, and this was amplified for some contrasts. A summary table showing of all our observations across the four MAE groups is presented below, and observations within WN, comparing AbEng listeners with MAE listeners in WN, are summarized in Table 6. We use these to guide us in returning back to the research questions.

As summarized in Table 5, for the MAE listeners we found significant differences depending on the age of the listeners for 3 of the 6 contrasts (all of the /e-æ/ contrasts) and regional variation for 4 of the 6 contrasts (all /e-æ/ contrasts and *hit-het*).

**Table 5.** Summary of Observations Across Sites, Predicted Merger Contrasts Labeled*.

Contrast	Observations re. 4 sites (MAE)
hell-Hal* (§ 4.1.1, Figure 3)	*Site-wise differences, age differences.*
	Agreement of endpoints of the continua for AW, and young MI. Older MI some merger behavior for *hell*. ML some evidence of merger in perception, more for older listeners. WN evidence for merger for young, answering at random at Step 7 (*Hal* is ambiguous), tendency to choose *hell*. Some merger behavior for older listeners at Step 1.
*Het-hat* (§ 4.1.2, Figure 5)	*Site-wise differences, age differences.*
	Agreement of endpoints of the continua for rural communities with some regional difference. Age differences most pronounced in WN. Urban listeners react differently, ML switch to *hat* sooner.
*hill-hell* (§ 4.1.3, Figure 7)	*No differences*
	Agreement of endpoints of the continua by all communities, only continuum step is significant.
*hit-het* (§ 4.1.4, Figure 9)	*Site-wise differences, no age differences*
	Urban and rural differences. Rural listeners hold onto *hit* longer; ML listeners switch to *het* soonest.
*mill-Mel* (§ 4.1.5, Figure 12)	*Site-wise differences, age differences.*
	Agreement of endpoints of the continua for all groups, only continuum step is significant. Nasal condition does not appreciably raise compared with *hill-hell*, although continuum step crosses over at Step 5 instead of Step 6 for rural listeners.
Mel-Mal* (§ 4.1.6, Figure 13)	*Site-wise differences, age differences.*
	(Almost) complete agreement of endpoints of the continua for MI young, ML young, AW young and old (no age difference within AW). Older MI stands apart, very strong preference for *Mal*, preference also seen for older ML and WN listeners to a degree (likely a lexical effect). WN merger for young, merger behavior at both ends of the continuum.

*Note.* MAE: Mainstream Australian English; AW: Albury-Wodonga; MI: Mildura; ML: Melbourne; WN: Warrnambool.

Within WN, we saw that there were often more *similarities* across the listener groups than we saw across the sites; in other words regional variation and age variation was often more pronounced than differences between the Aboriginal and non-Aboriginal listeners. Nevertheless, these results bring to mind comments discussed earlier, whereby *some* variability can become encoded in the system as sound change. The results also show that lexical frequency may be playing some role in how people perceive the boundary contrasts more generally, and that it may also be impacting listener groups differently as well, with the names *Mel-Mal* patterning strikingly differently for older and younger listeners.

With regard to our research questions, we can now address sociophonetic variation in the categorization of AusE vowels among the speaker groups. In terms of the geographical limits of the /el/-/æl/ merger (RQ1), results for *hell-Hal and Mel-Mal* showed that the /el/-/æl/ merger is indeed regionally conditioned; in perception, this merger occurs mostly in Warrnambool and is more entrenched for the Aboriginal listeners. In Melbourne and Mildura, older listeners had evidence of merger behavior which was not present for the younger groups, suggesting a possible reversal of merger in these locations. In contrast, listeners in the northern community of Albury-Wodonga have no merger in perception.

The results of our study are useful for showing the impact on perception of the wide category variability of the /e-æ/ distinction in AusE, and especially in prelateral environments. To determine which vowel they hear, listeners are relying on their experience—but in some cases they are unable to use this effectively because of exposure to spectrally unclear tokens (especially in the southern communities). We have seen this most clearly in the AbE group, for which we now have both perception (i.e., this study) and production data (Loakes et al., 2016). More focused phonetic analysis looking at the Australian participants’ vowel productions is currently underway to better understand all of the patterns we have observed.

As discussed in the introduction, Harrington and Schiel (2017) argue that sound change is inevitable, occurring when instability (variability) is present within communities. Our study on AusE perception shows that the identified variability (merger of /el/-/æl/) may in fact be *reducing* when looking at results in many of the communities, given that younger listeners are generally more able to distinguish /el/-/æl/ than older listeners. In this way, the merger cannot necessarily be analyzed as a completely Victorian sound change as such (and perhaps in some places, not a sound change at all). In Warrnambool, however, the story is somewhat different, with younger speakers showing clear merger behavior in perception, and merger behavior being even more entrenched for the Aboriginal listeners. In Warrnambool, the evidence may be pointing to an actual sound change, with merger in perception becoming stronger in the younger generation, although it would be prudent to analyze other phonetic contexts as well in future research, to make this claim more strongly.

This brings us to RQ2, which highlighted regional variability not previously understood in AusE. Focusing now on the MAE participants from the four locations, this study has given insight into the way in which listeners across Victoria categorize vowels more generally. Overall, there was some variation in response patterns depending on location; listeners in different communities had different crossovers from one phoneme category to the next in many of the contexts studied. This was seen most strongly in the /e/-/æ/ category, which we found to be generally more variable and less robust than the other contrasts. We know from past research that this matches production variability as well (i.e., Loakes et al., 2016, for the Warrnambool Aboriginal community). On reflection, this is also unsurprising given what we know about diachronic change in AusE vowels, where there is a very large amount of variation in the way speakers produce /e/-/æ/ (e.g., Cox, 1998, 1999; Cox & Palethorpe, 1998) and consequently in how they perceive the contrast (Mannell, 2004). In particular for the MAE listeners, this is supported in particular in the results for *het-hat* and *hit-het* contrasts, where the urban Melbourne listeners switched to the more open vowel category sooner than the rural listeners. The fact that the urban listeners switch sooner to a more open vowel supports the impression that vowel lowering is more pronounced in urban areas compared with rural areas. The variable distributions we have seen for listener responses across the four regions is similar to findings by Pinget et al. (2017, 2019), who concluded that regional variation they observed (in perception of consonantal changes) represented different stages of sound change. This is likely what we are observing in our study, which is supported by the urban/ rural patterning discussed above.

Research on other English varieties (i.e., not AusE) has shown that, in general, phonemic categories with high variance and overlapping distributions are considered to be “unstable”—listeners are faced with some processing costs in establishing clear targets, resulting in a lack of robustness in perception (i.e., Clopper, 2014, p. 75). In terms of the amount of regional variability seen in perception in this study; as mentioned above we saw that /ɪ/-/e/ was relatively invariable in perception across the MAE-speaking communities, with only continuum step being an important factor in response patterns for this contrast prelaterally. There were, however, some differences in *hit-het* with Melbourne listeners being particularly different from Albury-Wodonga in their responses, and the other rural communities patterning together for the most part. With Albury-Wodonga being so different from Melbourne, the data may suggest that people in this community pattern with Sydney to some degree. In some cases, Mildura also stood apart from the other sites (and especially the older listeners), which may hint at the fact that people in this region are influenced to some degree by speech patterns in South Australia and its capital Adelaide. Given the fact that both Mildura and Albury-Wodonga are situated on the northern border of Victoria, and given the distance to resources (especially with Adelaide being far closer to Mildura compared with Melbourne), this is not an unlikely scenario. Further research will look at the way that people in Mildura and Albury-Wodonga pattern compared with urban centers outside Victoria, in particular Sydney and Adelaide.

Our third research question focused on how L1 AbE listeners categorized MAE vowels. As mentioned, we saw that both listener groups in Warrnambool had similar trends in responses, but we still found significant differences in perception between AbEng and MAE perceptual behavior for 5 of the 6 contrasts, excluding *hill-hell*. Again, the differences seen across the Aboriginal and MAE speakers appear to be directly linked to production factors. In other words, accent differences between mainstream and Aboriginal listeners shape categorization performance. When speaking, these AbE listeners use “higher” vowels than MAE speakers and when listening to the mainstream speaker they essentially hold on to the phonetically higher vowel for longer. As mentioned, there may also be different lexical frequency effects between the two different Warrnambool communities.

As summarized in Table 6, with respect to age in Warrnambool, we also saw that the differences were more pronounced between the older Aboriginal and non-Aboriginal groups, which may well reflect the fact that the younger listeners from both communities tend now to have more similar experiences in input. This may be because the younger Warrnambool group have more education, and more mobility within the community in which they live compared with the older Warrnambool listeners.^5^

**Table 6.** Summary of Observations Within Warrnambool (Across Communities), Predicted Merger Contrasts Labeled*.

Contrast	Observations re. community (within WN)
hell-Hal* (§ 4.1.1, Figure 4)	*Community differences, age differences*.
	MAE and AbEng listeners have merger behavior, and amongst the young in particular (tendency to choose *hell*). Within communities, age differences between MAE listeners is most marked.
*Het-hat* (§ 4.1.2, Figure 6)	*Community differences, no age differences.*
	Very similar overall patterns when compared with site-wise observations, but significant differences emerge for community, not age. Crossover to *het* happens early.
*hill-hell* (§ 4.1.3, Figure 8)	*Age differences, no community differences.*
	AbEng and MAE respond in the same way. Age differences within WN, younger listeners (of both varieties) tend to choose *hill* regardless of step. Young have less variability in responses.
*hit-het* (§ 4.1.4, Figure 10)	*Community differences, no age differences.*
	Similar response patterns overall for WN, but MAE switch to *het* sooner.
*Mill-Mel* (§ 4.1.5, Figure 12, also reported in text)	*Community differences, age differences*
	Tendency to choose *mill* for both AbEng age groups, and for younger WN listeners. Overall categorical perception, nasal does not have expected effect.
Mel-Mal* (§ 4.1.6, Figure 14.)	*Community differences, age differences.*
	Merger behavior evident for AbEng and younger MAE. Significant differences between AbEng and MAE, difference strongest between older groups. Age differences greater for MAE. Younger listeners in both groups tend to choose *Mel*, older listeners tend to choose *Mal*.

*Note.* WN: Warrnambool; MAE: Mainstream Australian English; AbE: Aboriginal English.

RQ4 looked specifically at age, and we showed that this was a significant factor in response patterning, particularly for perception of /e-æ/. Age differences were observed across both MAE and AbEng listener groups, but this was strongest for MAE listeners and especially for Warrnambool. This accords with previous research showing that listeners are exposed to a large amount of production variability in their communities, especially due to age-related speech patterning whereby older speakers have “higher” vowels (e.g., Cox, 1999; Cox & Palethorpe, 2008; Mannell, 2004). It also accords with research showing that AbE listeners are not taking part in short front vowel lowering to the same extent as MAE groups (Loakes et al., 2016). Our perception findings both extend and support earlier work by Mannell (2004) which showed a high degree of perceptual variability in perceptual categorization of /e/.

To summarize, we showed in this study that AusE vowel perception does indeed vary sociophonetically (cross-regionally, across age groups, and across varieties). The study shows that variation in AusE is not only confined to the production system, and is in fact also associated with perception behavior, as has been seen for vowels in other varieties (e.g., Kendall & Fridland, 2012 for American English; Janson, 1983 for Swedish; and Brunellière et al., 2009 for French) as well as consonants (Pinget et al., 2017, 2019 for Dutch). Response patterns (for age and variety in particular) align with the types of variability that have been observed in the AusEng vowel system in previous research. We also see that a vowel merger for /el-æl/ patterns in a more complex way than previously understood, being associated with older listeners in some regions and younger listeners in another. As discussed throughout, lexical frequency effects are also a consideration in some of the patterns observed. The findings show that this merger may be reversing; in most communities the younger speakers distinguish /el-æl/, however, Warrnambool was a different case with far more entrenchment for the younger group. This is a particularly interesting finding, highlighting the unexpected volatility of phonetic processes, which are effectively moving in different directions in different locations and presumably causing randomness and a lack of predictability for listeners.

The results of this study support models showing that listening and sound change are experienced-based (i.e., Norris & McQueen, 2008; Ohala, 1989, 1991, 1993a, 1993b; Pinget et al. 2017, 2019; Warren et al., 2007; Warren & Hay, 2005). Despite the fact that AusE has far less regionally determined phonetic variability than other English varieties spoken across large geographical areas, we nevertheless observed regional differences among listeners in our study. We saw evidence that listeners choose exemplars which best suit what they regularly hear in input—and we can gauge community input in some of the studied locations based on previous production research involving the same speaker-listeners (Loakes, Clothier, et al., 2014 for Warrnambool, and Loakes et al., 2016 for the AbE sample). This assertion is also supported by similar research on variability in perception in other communities (e.g., Kendall & Fridland, 2012).

The question remains as to why we should see differing rates of change (and general variability) across the communities. In their IP model, Harrington et al. (2018) note that instability and asymmetry are factors, and we have seen this in the current study, but it is also *individuals* and the unique linguistic circumstances they find themselves in, which are responsible for this (also supported in work by Baker et al., 2011; Voeten, 2021). Harrington et al. (2018) state:

. . .whether or not sound change actually comes about depends upon which speakers regularly speak to each other, whether a phonetic bias happens to be magnified by interaction, and whether or not sub-phonemic classes are fragmented and regrouped over time. . . . These probabilities in combination also explain why spoken accents . . . and languages are phonetically so idiosyncratic. (p. 17)

This indicates the need for tracking individual behavior, an important next step in further understanding the reasons behind the variability we have observed here. Early results (Loakes et al., 2019) show remarkable consistency among listeners over a 6-year time span, but it will be important to focus on large amounts of data from individuals’ production and perception to better understand the forces at play.

Overall, this study has made some headway in understanding how sociophonetic variation plays out in perception for AusE listeners. By including AbE listeners in the study we have shown how useful it is analyze regionality and social characteristics in the same study. We saw socially differential behavior among the two groups of speakers in the same regional area (Warrnambool), and likewise we also saw clearly regional behavior among MAE listeners who live in different locations. While this study has uncovered some previously unknown sociophonetic variability in AusE regarding the perceptual categorization of vowel sounds, it has also opened up further questions. In future work, we will look more closely at the alignment of production and perception in some of the groups discussed here, as well as further delve into individual behavior. Finally, comparative analysis of another AbE listener group from Mildura is also underway, in order to understand whether regional variability in perception exists among AbE listeners, and whether varietal differences exist in that location.

## Appendix

**Table A1.** Mid-Point Formant Frequencies of Real Speech Tokens Used in Stimulus Creation.

Word	F1 (Hz; steady state)	F2 (Hz; steady state)	F3 (Hz; steady state)
*hit*	426	2,874	3,355
*hill*	470	2,398	3,313
*mill*	400	2,440	2,640
*Het*	562	2,317	2,907
*hell*	683	2,026	2,562
*Mel*	571	2,002	2,781
*hat*	1,004	2,068	3,015
*Hal*	1,085	1,742	2,993
*Mal*	1011	1,642	2,414

**Table A2.** Model Summary for Hell-Hal Responses (Within MAE).

Predictors	Odds ratios	CI	*z-*statistic	*p*
(Intercept)	624.61	[81.36, 4,795.11]	6.19	<.001
step	0.21	[0.13, 0.32]	–6.98	<.001
site [MI]	0.03	[0.0, 0.43]	–2.60	.009
site [ML]	0.01	[0.0, 0.13]	–3.66	<.001
site [WN]	0.03	[0.0, 0.37]	–2.73	.006
age.grp [younger]	5.58	[0.25, 125.21]	1.08	.278
step × site [MI]	1.72	[0.97, 3.05]	1.87	.062
step × site [ML]	2.86	[1.76, 4.65]	4.23	<.001
step × site [WN]	1.89	[1.08, 3.31]	2.22	.026
step × age.grp [younger]	0.62	[0.31, 1.26]	–1.31	.190
site [MI] × age.grp [younger]	31.95	[0.41, 2,479.17]	1.56	.119
site [ML] × age.grp [younger]	1.18	[0.03, 40.91]	0.09	.929
site [WN] × age.grp [younger]	0.44	[0.01, 18.20]	–0.44	.663
(step × site [MI]) × age.grp [younger]	0.53	[0.20, 1.46]	–1.22	.222
(step × site [ML]) × age.grp [younger]	1.13	[0.52, 2.42]	0.31	.759
(step × site [WN]) × age.grp [younger]	2.39	[1.05, 5.41]	2.08	.037
Observations	1,389			
Marginal *R*^2^/Conditional *R*^2^	.650/.741			

*Note.* Final model: response ~ (1 − listener) + step + site + age.grp + step: site + step:age.grp + site:age.grp + step: site:age.grp. MAE: Mainstream Australian English; CI: confidence interval; MI: Mildura; ML: Melbourne; WN: Warrnambool.

**Table A3.** Regression Model Results for Hell~Hal Perception for Warrnambool Listeners.

Predictors	Odds ratios	CI	z-statistic	*p*
(Intercept)	52.6	[10.86, 254.67]	4.92	<.001
step	0.48	[0.38, 0.62]	–5.61	<.001
variety [MAE]	0.42	[0.04, 3.97]	–0.76	.447
age.grp [younger]	0.67	[0.11, 4.19]	–0.43	.668
step × variety [MAE]	0.75	[0.49, 1.17]	–1.27	.205
step × age.grp [younger]	1.38	[1.03, 1.84]	2.17	.03
variety [MAE] × age.grp [younger]	2.84	[0.17, 47.33]	0.73	.466
(step × variety [MAE]) × age.grp [younger]	1.13	[0.68, 1.88]	0.46	.645
Observations	912			
Marginal *R*^2^/Conditional *R*^2^	.328/.530			

*Note.* Final model: response ~ (1 − listener) + step + variety + age.grp + step: variety + step:age.grp + variety:age.grp + step: variety:age.grp. CI: confidence interval; MAE: Mainstream Australian English.

**Table A4.** Regression Model Summary for Het-Hat Continuum for MAE Listeners.

Predictors	Odds ratios	CI	*z*-statistic	*p*
(Intercept)	9697.35	[329.73, 285,200.15]	5.32	<.001
step	0.10	[0.05, 0.21]	–6.11	<.001
site [MI]	0.02	[0.0, 1.36]	–1.82	.069
site [ML]	0.0	[0.0, 0.12]	–3.01	.003
site [WN]	0.12	[0.0, 19.42]	–.81	.416
age.grp [younger]	0.02	[0.0, 0.99]	–1.96	.050
step × site [MI]	1.55	[0.57, 4.18]	0.86	.392
step × site [ML]	2.63	[1.14, 6.09]	2.26	.024
step × site [WN]	0.40	[0.08, 1.93]	–1.14	.254
step × age.grp [younger]	2.03	[0.89, 4.63]	1.69	.092
site [MI] × age.grp [younger]	842.03	[2.94, 241,299.62]	2.33	.020
site [ML] × age.grp [younger]	163.18	[1.21, 21,973.42]	2.04	.042
site [WN] × age.grp [younger]	2.08	[0.01, 686.89]	.25	.805
(step × site [MI]) × age.grp [younger]	0.32	[0.09, 1.16]	–1.73	.083
(step × site [ML]) × age.grp [younger]	0.25	[0.08, 0.78]	–2.40	.016
(step × site [WN]) × age.grp [younger]	3.56	[0.68, 18.79]	1.50	.134
Observations	,442			
Marginal *R*^2^/Conditional *R*^2^	.705/.876			

*Note.* Final model: response ~ (1 − listener) + step + site + age.grp + step: site + step:age.grp + site:age.grp + step: site:age.grp. MAE: Mainstream Australian English; CI: confidence interval; MI: Mildura; ML: Melbourne; WN: Warrnambool.

**Table A5.** Regression Model Summary for Het~Hat Continuum for Warrnambool Listeners.

Predictors	Odds ratios	CI	*z*-statistic	*p*
(Intercept)	0.16	[0.07, 0.36]	–4.42	<.001
step	0.35	[0.29, 0.42]	–10.91	<.001
variety [MAE]	0.89	[0.25, 3.24]	–0.18	.861
step × variety [MAE]	0.69	[0.48, 1.00]	–1.99	.047
Marginal *R*^2^/Conditional *R*^2^	.481/.729			

*Note.* Final model: response ~ (1| listener) + step + variety + variety:step. CI: confidence interval; MAE: Mainstream Australian English.

**Table A6.** Regression Model Summary for Hill-Hell Continuum Within MAE Listeners.

Predictors	Odds ratios	CI	*z*-statistic	*p*
(Intercept)	40,778.92	[9,734.40, 170,829.27]	14.52	<.001
step	0.12	[0.09, 0.16]	–15.72	<.001
Observations	1,417			
Marginal *R*^2^/Conditional *R*^2^	.700/.870			

*Note.* Final model: response ~ (1 − listener) + step. MAE: Mainstream Australian English; CI: confidence interval.

**Table A7.** Summary Statistics for Hill-Hell Responses by Age Group for Warrnambool Listeners.

Age group	Hell		Hill		Sum	
	Count	%	Count	%	Count	%
Older	106	37.19	179	62.81	285	100
Younger	182	28.48	457	71.518	639	100

**Table A8.** Regression Model Summary for Hill~Hell Perception for Warrnambool Listeners.

Predictors	Odds ratios	CI	*z*-statistic	*p*
(Intercept)	5.26	[1.90, 14.54]	3.2	.001
step	0.15	[0.11, 0.20]	–13.09	<.001
age.grp [younger]	3.95	[1.16, 13.42]	2.2	.028
Observations	1,417			
Marginal *R*^2^/Conditional *R*^2^	.700/.870			

*Note.* Final model: response ~ (1 − listener) + step + age.grp. CI: confidence interval.

**Table A9.** Regression Model Summary for Hit~Het Perception for MAE Listeners.

Predictors	Odds ratios	CI	*z*-statistic	*p*
(Intercept)	29,773.58	[2,154.04, 411,536.85]	7.69	<.001
step	0.17	[0.11, 0.26]	–8.42	<.001
site [MI]	1.01	[0.03, 41.10]	0.01	.994
site [ML]	0.02	[0.0, 0.51]	–2.39	.017
site [WN]	1.01	[0.03, 38.23]	0.01	.996
step × site [MI]	0.75	[0.41, 1.39]	–0.91	.363
step × site [ML]	1.40	[0.86, 2.28]	1.37	.172
step × site [WN]	0.77	[0.42, 1.40]	–0.85	.395
Observations	1,444			
Marginal *R*^2^/Conditional *R*^2^	.604/.861			

*Note.* Final model: response ~ (1 − listener) + step + site + step:site. MAE: Mainstream Australian English; CI: confidence interval; MI: Mildura; ML: Melbourne; WN: Warrnambool.

**Table A10.** Regression Model Summary for Hit~Het Perception for Warrnambool Listeners.

Predictors	Odds ratios	CI	*z*-statistic	*p*
(Intercept)	1,149.76	[257.76, 5,128.70]	9.24	<.001
step	0.29	[0.23, 0.36]	–10.72	<.001
variety [MAE]	11.29	[0.74, 172.24]	1.74	.081
step × variety [MAE]	0.53	[0.33, 0.84]	–2.69	.007
Observations	916			
Marginal *R*^2^/Conditional *R*^2^	.545/.803			

*Note.* Final model: response ~ (1 − listener) + step + variety + step:variety. CI = confidence interval; MAE: Mainstream Australian English.

**Table A11.** Regression Model Summary for Mill-Mel Perception for MAE Listeners.

Predictors	Odds ratios	CI	*z*-statistic	*p*
(Intercept)	51,961.16	[12,362.62, 21,8397.18]	14.82	<.001
step	0.09	[0.06, 0.12]	–15.63	<.001
Observations	1,448			
Marginal *R*^2^/Conditional *R*^2^	.811/.890			

*Note.* Final model: response ~ (1 − listener) + step. MAE: Mainstream Australian English; CI = confidence interval.

**Table A12.** Summary Statistics for Mill-Mel Responses in Warrnambool by Age.

Age group	Mel		Mill		Sum	
	Count	%	Count	%	Count	%
Older	131	45.64	156	54.36	287	100
Younger	230	36.62	398	63.38	628	100

**Table A13.** Summary Statistics for Mill-Mel Responses in Warrnambool by Variety.

Variety	Mel		Mill		Sum	
	Count	%	Count	%	Count	%
AbE	212	36.3	372	63.7	584	100
MAE	149	45.02	182	54.98	331	100

*Note.* AbE: Aboriginal English; MAE: Mainstream Australian English.

**Table A14.** Regression Model Summary for Mill-Mel Perception for Warrnambool Listeners.

Predictors	Odds ratios	CI	*z*-statistic	*p*
(Intercept)	1,539.87	[487.26, 4,866.39]	12.50	<.001
Step	0.19	[0.15, 0.24]	–14.57	<.001
Variety [MAE]	0.37	[0.19, 0.71]	–3.01	.003
Age.grp [younger]	2.96	[1.51, 5.78]	3.17	.002
Observations	915			
Marginal *R*^2^/Conditional *R*^2^	.754 /.781			

*Note.* Final model: response ~ (1 − listener) + step + variety + age.grp. CI: confidence interval; MAE: Mainstream Australian English.

**Table A15.** Regression Model Summary for Mel-Mal Perception for MAE Listeners.

Predictors	Odds ratios	CI	*z*-statistic	*p*
(Intercept)	161.80	[28.61, 914.93]	5.75	<.001
Step	0.22	[0.14, 0.33]	–6.95	<.001
Site [MI]	0.0	[0.0, 0.03]	–5.15	<.001
Site [ML]	0.03	[0.0, 0.21]	–3.43	.001
Site [WN]	0.02	[0.0, 0.21]	–3.32	.001
Age.grp [younger]	3.86	[0.31, 48.47]	1.05	.296
Step × site [MI]	3.53	[2.13, 5.85]	4.90	<.001
Step × site [ML]	2.02	[1.20, 3.39]	2.65	.008
Step × site [WN]	1.85	[1.04, 3.29]	2.09	.037
Step × age.grp [younger]	0.86	[0.47, 1.57]	–0.49	.621
Site [MI] × age.grp [younger]	284.64	[8.51, 9,521.87]	3.16	.002
Site [ML] × age.grp [younger]	3.14	[0.15, 65.80]	0.74	.462
Site [WN] × age.grp [younger]	0.51	[0.02, 11.34]	–0.42	.674
(Step × site [MI]) × age.grp [younger]	0.24	[0.10, 0.55]	–3.35	.001
(Step × site [ML]) × age.grp [younger]	1.02	[0.50, 2.07]	0.04	.965
(Step × site [WN]) × age.grp [younger]	2.01	[0.95, 4.22]	1.83	.067
Observations	1440			
Marginal *R*^2^/Conditional *R*^2^	.613/.711			

*Note.* Final model: response ~ (1 − listener) + step + site + age.grp + step: site + step:age.grp + site:age.grp + step: site:age.grp. MAE: Mainstream Australian English; CI: confidence interval; MI: Mildura; ML: Melbourne; WN: Warrnambool.

**Table A16.** Regression Model Summary for Mel~Mal Perception for Warrnambool Listeners.

Predictors	Odds ratios	CI	*z*-statistic	*p*
(Intercept)	3.20	[0.99, 10.40]	1.94	.053
Step	0.68	[0.57, 0.82]	–4.10	<.001
Variety [MAE]	1.09	[0.17, 6.94]	0.09	.926
Age.grp [younger]	3.09	[0.74, 12.81]	1.55	.121
Step × variety [MAE]	0.58	[0.38, 0.90]	–2.46	.014
Step × age.grp [younger]	1.04	[0.84, 1.29]	0.34	.736
Variety [MAE] × age.grp [younger]	0.68	[0.07, 6.80]	–0.32	.746
(Step × variety [MAE]) × age.grp [younger]	1.66	[1.02, 2.69]	2.05	.041
Observations	917			
Marginal *R*^2^/Conditional *R*^2^	.332/.514			

*Note.* Final model: response ~ (1 − listener) + step + variety + age.grp + step: variety + step:age.grp + variety:age.grp + step: variety:age.grp. CI: confidence interval; MAE: Mainstream Australian English.

## Notes

1. Thanks are due to the late Anne Cutler for bringing this to our attention.

2. While the singular “Aboriginal English” is used here, it is important to acknowledge Diana Eades’ (2013) use of the phrase “Aboriginal ways of using English,” which better reflects the variation found across Australia. In this current paper, *Aboriginal English* will be appropriate for talking about a single community in the southern part of Australia, but “Aboriginal Englishes” better reflects the situation across different communities in Australia as a whole.

3. F1, F2, and F3 taken at the steady state of the vowel to give a general indication of how the vowels were realized. Values are also provided for F3, which, while not essential to perceptual identification of most vowels, including /e/–/æ/, by English speakers, is known to facilitate it (Hillenbrand et al., 1995, p. 3109). While F3 is essentially the same in *Hal, het* and *hat*, it is much lower in *hell*—a possible indication of greater lip-rounding and tongue dorsum raising in the adjacent velarized lateral in *hell* compared with *Hal*.

4. We attempted including random slopes by listener in our models, which would control for individual differences in listener responses to the differences in continuum steps; however, these more complex models failed to converge.

5. This information has come from participant background questionnaires and from general discussion with the interviewer about their lives and activities.
